# Semi-biological photosystem: harnessing carbon dots and *Geobacter sulfurreducens* for solar-driven hydrogenation

**DOI:** 10.1093/sumbio/qvae020

**Published:** 2024-07-22

**Authors:** Mandy Ching Man Yau, Shafeer Kalathil

**Affiliations:** Hub for Biotechnology in the Built Environment, Department of Applied Sciences, Faculty of Health and Life Sciences, Northumbria University, Newcastle NE1 8ST, United Kingdom; Hub for Biotechnology in the Built Environment, Department of Applied Sciences, Faculty of Health and Life Sciences, Northumbria University, Newcastle NE1 8ST, United Kingdom

**Keywords:** environmental microbiology, microbial biotechnology, renewable, nanoparticles, soil microbes

## Abstract

Semi-biological photosynthesis utilizes the unique ability of microbial catalysts together with synthetic photosensitizers (semiconductors) to produce high-value chemicals from sustainable feedstocks. In this work, we devise a semi-biological hybrid system consisting of sustainable photosensitizers, carbon dots in the size range of 5–35 nm (CDs) interfaced with bacteria, *Geobacter sulfurreducens*, to reduce fumarate to succinate as a model hydrogenation reaction. After 7 days of solar irradiation, using quantitative proton nuclear magnetic resonance spectroscopy (qNMR), the CD−*G. sulfurreducens* photosystem produced ∼18 mM of succinate without the need for a redox mediator. Moreover, in reusing the CDs, ∼70% of the succinate (compared to the previous cycle) was recovered. The proposed photobiohybrid system paves a new avenue for sustainable solar-to-chemical conversion in high-value chemical production.

**Sustainability Statement**

Solar energy holds significant potential in addressing the growing energy demands while also mitigating carbon emissions. The immediate products obtained from carbon dioxide reduction are currently unable to compete with fossil fuels. Hence, we developed a system where carbon dots were integrated with *Geobacter sulfurreducens* for solar-driven hydrogenation of fumarate to succinate, a key intermediate in bio-based production, which can be used in subsequent biochemical processes to make higher-value products. Beyond succinate production, our semi-biological hybrid photosystem proves to be a promising platform for the sustainable synthesis of higher-value compounds such as 1,4-butanediol and N-methyl-2-pyrrolidone, the former being used in manufacture of plastics and the latter in the fabrication of lithium-ion batteries and removal of pains, just to name a few. The Sustainable Development Goals (SDGs) of the UN are met by our work: affordable and clean energy (SDG 7) and climate action (SDG 13).

## Introduction

Artificial photosystems can use solar energy to drive reactions to produce hydrogen from water or to reduce carbon dioxide (CO_2_). However, the current bottlenecks involved in CO_2_ utilization include (but are not limited to): low efficiencies and selectivity, energetic demand, low atom economy, inability to form higher density fuels, and inertness of CO_2_, all of which impede the deployment of carbon transformation technologies on a large scale (Yang et al. 2012, Artz et al. 2018). Moreover, while it has been observed that traditional artificial photosystems display high solar to fuel efficiency compared to natural photosystems (Boardman et al. 1980, Fusco et al. 2022, Ghosh et al. 2022) (with photovoltaic cells reaching to >20% solar-to-electricity conversion rates) (Jia et al. 2016, Zheng et al. 2023), artificial photosystems struggle to produce high-value multi-carbon products such as n-propanol (Klabunde 2018, Propanol Market Size, Share, Trends & Growth Report, 2023–2030. 2023). On the other hand, semi-biological hybrid photosystems introduce a biological component, typically either enzymes or microbes, to work alongside the artificial light absorber by carrying out the desired chemical transformation (Fang et al. 2020, Yau et al. 2022). The high selectivity and rates presented by enzymes in semi-biological photosystems are very attractive. Successful solar fuel production has been achieved with enzymes interfaced with artificial light absorbers (Yadav et al. 2012, Mersch et al. 2015, Holá et al. 2020). However, as a whole, enzymes are very delicate biocatalysts that are susceptible to changes in pH and temperature, and the enzyme isolation and purification may prove laborious depending on the enzyme and whether they are available commercially (Bisswanger 2008). For these reasons, it is important to consider the advantages and disadvantages when interfacing artificial and biological components, which is where the power of microbes can be displayed.

Using microbes instead of enzymes in semi-biological hybrid photosystems overcome the limitations presented by the enzymatic and artificial components by compensating for each component’s aforementioned shortcomings. Microbes while only first discovered in the 1600s (Gest 2004), have existed for millions of years and have evolved to have extremely efficient metabolic pathways (Meléndez-Hevia et al. 1996). Unlike enzymes, not only they are more forgiving towards environmental changes (e.g. pH or temperature), but they are readily available to convert sustainable feedstocks (e.g. waste carbon, organic molecules) to more complex products by utilizing their inherent biochemical pathway to make a variety of complex biochemicals. The tricarboxylic acid cycle (TCA) is one of such biochemical reaction pathways present in many microorganisms and has been extensively studied (Gadd and Kim 2019). On the flip side, the reverse TCA cycle also exists in microbes and is able to uptake adenosine triphosphate (ATP) and CO_2_ to produce longer-chained organic molecules (Choi et al. 2022). The reduction of fumarate to succinate in particular, has become a model reaction for studying photocatalysis and photoreduction reactions (Zhou and Guzman 2016, Mangiante et al. 2019). In this work, fumarate reduction was chosen over CO_2_ because the immediate, higher-value products produced are more competitive than those made from the reduction of CO_2_ (often limited to carbon monoxide, formate, or other short-chained carbon molecules) (Jhong et al. 2013, Lu and Jiao 2016) in that they can act as a segue to the formation of other high-value biofuels. In addition, thermodynamically, even one electron reduction of CO_2_ has a high energy cost [E^0^ = −1.85 V vs. standard hydrogen electrode (SHE) at pH 7] (White et al. 2015). Thus, implementing microbes with artificial photocatalysts will overcome the sensitivity presented by enzymes and the inability to produce high-value products by artificial photosystems alone.

Succinate is an important chemical used in many food and pharmaceutical industries (Zeikus et al. 1999). At present, it can be synthesized through hydrogenation of maleic anhydride or maleic acid (Bellardita et al. 2023) using fossil fuels, but the solar-driven reduction of fumarate to succinate has also been previously achieved through the use of fumarate reductase coupled with carbon dots (CDs) (Hutton et al. 2016). However, the instability of the system was made apparent when the rate of reaction started to decrease after only 2 h due to the degradation of the enzyme. Likewise, this instability is also shown in the work conducted by Bachmeier et al. (2014), where the enzyme flavocytochrome C_3_ was immobilized onto ruthenium bipyridine (RuP)-modified titanium dioxide and RuP-modified zirconium dioxide nanoparticles. While succinate has also been derived from CO_2_ in a hybrid approach, it is a two-stage process where CO_2_ is first converted chemically to formate/formic acid before microbially transforming to succinic acid via *Mannheimia succinicproducens* (Ahn et al. 2017). Additionally, the microbe used had to be first engineered to uptake formic acid efficiently (Ahn et al. 2017). In our hybrid photosystem, fumarate reduction is taken place in, essentially, a one-pot system by *G. sulfurreducens* readily.

In this report, *G. sulfurreducens* was used for the solar-driven reduction of fumarate to succinate in the presence of CDs (semiconductors). CDs are carbon-based nanoparticles with favourable attributes like having a tuneable bandgap (Choi et al. 2018, Mehta et al. 2019) and contain UV-vis light absorption properties that extend into near-infrared spectral range, thus covering a large portion of the solar spectrum (Fernando et al. 2015, Li et al. 2018). Moreover, although CDs are considered low in terms of toxicity (Wang et al. 2014, Park et al. 2020), it is dependent on the target and what it is made out of. For example, metals such as silver (Lashin et al. 2021) and gold (Zhao et al. 2010) have been extensively studied with regards to their anti-microbial properties. In the study conducted by Walia et al. (2019b), their CDs could induce oxidative stress in *Escherichia coli* and *Staphylococcus aureus*, resulting in DNA degradation (Walia et al. 2019b). All in all, although the functionalities are different, CDs can exhibit anti-microbial traits (Varghese and Balachandran 2021). Therefore, to avoid microbial death or inhibition of fumarate reduction, the CDs chosen in our system were made with non-toxic citric acid and were free from metals to avoid risk of anti-microbial activity.

*Geobacter sulfurreducens* is an ideal candidate for making succinate in a semi-artificial hybrid photosystem due to its innate ability to respire on fumarate to produce succinate (Galushko and Schink 2000). Unlike other succinate-producing microorganisms like *Shewanella oneidensis* and *Mannheimia succiniciproducens* (Lee et al. 2002), *G. sulfurreducens* expresses heme containing c-type cytochromes and electrically conductive protein nanowires (e-PNs) (Lovley 2017), which help them to transport metabolically generated electrons in micrometre distances. e-PNs are composed of monomeric units of c-type cytochromes rich with heme (Fe) groups (Wang et al. 2019). In this work, *G. sulfurreducens* was coupled with CDs to convert fumarate to succinate. CDs are typically made up of sp^2^ (Yang et al. 2012) and sp^3^ (Boardman et al. 1980) hybridized carbon atoms and are 1–10 nm in diameter (Zhu et al. 2015). Compared to traditional semiconductors that employ metals like gallium, titanium, and platinum, CDs can be made rather inexpensively using cheaper precursors like citric acid or urea (Hutton et al. 2016, Simões et al. 2016, Kasprzyk et al. 2018), or even food like watermelon (Zhou et al. 2012) and coffee (Jiang et al. 2014, Zhang et al. 2018). They have attracted a lot of interest due to their biocompatibility, stability, and photoluminescent properties (Zhao et al. 2008, Tao et al. 2012, Sendão et al. 2019), and their light-harvesting characteristics have promising prospects as a photosensitizer in a semi-biological hybrid photosystem. This study focuses solely on the integration between CDs and *G. sulfurreducens* to show successful reduction of fumarate to succinate. In addition, we show that a reasonable amount of succinate can be recovered after reusing the CDs from a previous iteration, which, to our belief, is the first for this particular semi-biological hybrid photosystem.

## Materials and methods

### Synthesis of CDs

Carboxylic-capped CDs (CD–CO_2_H) were synthesized as previously reported (Martindale et al. 2015). Citric acid (50 g) was heated at 180°C for 40 h. CD–CO_2_H were then centrifuged at 10 000 g for 20 min before dissolving with dH_2_O (distilled water). It was then frozen for 1 day at −80°C before dipping in liquid nitrogen for 10 s and freeze-drying.

### Culturing of *G. sulfurreducens*

*Geobacter sulfurreducens* PCA was obtained from the German Collection of Microorganisms and Cell Cultures (DSMZ). The strain was cultured in DSMZ 826 medium consisting of ammonium chloride, sodium phosphate dibasic, sodium bicarbonate, potassium chloride, trace element solution (Supplementary Table S1), and vitamin solution (Supplementary Table S2). A volume of 50 mM sodium fumarate (electron acceptor) and 20 mM sodium acetate (electron donor) were added to the medium and purged with a gas mixture containing N_2_:CO_2_ (80%:20%) for 45 min. Then *G. sulfurreducens* was inoculated into the serum vials and incubated at 30°C, 100 rpm for 1 week. The growth of *G. sulfurreducens* was monitored by measuring optical density (OD_600_) using a UV-vis spectrometer.

### Assembly of CDs and *G. sulfurreducens* for photoexperiments

A photovial containing the microbial medium (Supplementary Table S3), 20 mM sacrificial hole scavenger, triethanolamine (TEOA), 50 mM fumarate, and 1.5 mg/mL CD–CO_2_H was prepared in a photovial and sealed with a septa seal. The grown *G. sulfurreducens* were centrifuged at 4500 rpm for 10 min and washed with distilled H_2_O. The supernatant was removed and the residual *G. sulfurreducens* was washed and centrifuged twice further with DSMZ 826 growth media with sodium fumarate and sodium acetate omitted. The photovials were then purged with N_2_:CO_2_ (80%:20%) for 45 min. *Geobacter sulfurreducens* was then inoculated into the photovial until the optical density was 0.70. For the light experiment, the sample was then irradiated using a solar light simulator (AM 1.5 G, 1 sun, 100 mW cm^−2^). For the dark reactions, the vial was covered in tin foil. The experiment was carried out for 7 days at room temperature with a gentle stirring (pH:7).

### Physical characterization

The morphologies and sizes of CDs and *G. sulfurreducens* were characterized using scanning electron microscopy (SEM) (Tescan Mira 3).

### CD preparation for SEM

CDs (0.5 mg) were dispersed in 1 mL MeOH. Three drops were added to the specimen stub using a Pasteur pipette. The CDs were then coated in chromium (5 nm thickness).

### *Geobacter sulfurreducens* and CD−*G. sulfurreducens* hybrid preparation for SEM

*Geobacter sulfurreducens* (1 mL) was centrifuged at 7000 rpm for 2 min and washed with dH_2_O twice. The specimen was then fixed with 2.5% glutaraldehyde in sodium cacodylate buffer (0.20 M, pH 7.4) overnight. The solution was rinsed with sodium cacodylate buffer (0.20 M, pH 7.4) thrice for 10 min. The sample was then mounted onto the carbon tape-covered SEM stub. The sample was then dehydrated using increasing concentrations of ethanol (20, 30, 50, 70, and 100%), waiting 10 min in between each dehydration step and drying the sample twice with 100% ethanol. The specimen was then sputter coated with chromium (5 nm thickness) before it was ready to be imaged using the SEM. For CD−*G. sulfurreducens*, 1 mL was taken from the photovial after 7 days of solar irradiation. The solution was centrifuged at 7000 rpm for 10 min and washed with dH_2_O twice. The specimen was then fixed with 2.5% glutaraldehyde in sodium cacodylate buffer (0.20 M, pH 7.4) overnight. The solution was rinsed with sodium cacodylate buffer (0.20 M, pH 7.4) thrice for 10 min before being mounted onto the carbon tape-covered SEM stub. The sample was then dehydrated using increasing concentrations of ethanol (20, 30, 50, 70, and 100%), waiting 10 min in between each dehydration step and drying the sample twice with 100% ethanol. The specimen was then sputter coated with chromium (5 nm thickness) before it was ready to be imaged using the SEM.

The transmission electron microscopy (TEM) analysis was carried out using a Thermo Scientific Talos F200X G2 TEM (FEI, operating voltage 200 kV) to visualize e-PNs emanating from *G. sulfurreducens* cells. X-ray photon spectroscopy (XPS) of CDs was carried out on Shimadzu Kratos. X-ray diffraction (XRD) profiles for CDs were obtained with Rigaku SmartLab SE XRD with a copper source for the generation of X-rays. UV-visible absorption spectra of the CDs and *G. sulfurreducens* were taken using a Cary 50 Bio UV-visible spectrophotometer. CD–CO_2_H (1.5 mg) was dissolved in 1 mL dH_2_O and placed in a microcuvette with a path length of 1 cm. Fourier transform infrared spectroscopy (FTIR) spectra data of CDs were obtained using a Bruker Alpha FTIR spectrometer. CD–CO_2_H (1 mg) powder was placed on the crystal and compacted using the level ready for analysis.

### Quantification of succinate production using NMR

NMR analysis was performed on a JEOL ECS spectrometer at 400 MHz to track fumarate to succinate conversion. The NMR spectra obtained were then processed with Delta. The reaction mixture (1 mL) was extracted from the photovial using a syringe and filtered using a 0.2 µm syringe filter. A volume of 5 mM TSP in D_2_O was added to the solution (60 µL) as the internal standard. The solution was vortexed for 5 s before being transferred to an NMR tube.

## Results and discussion

### Synthesis and characterization of CD–CO_2_H

**Scheme 1.**
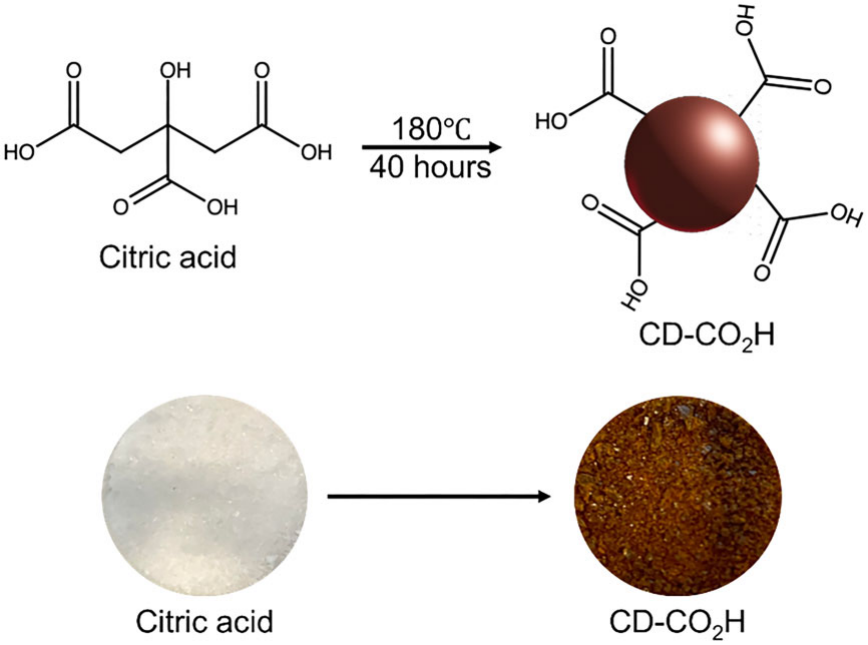
Synthesis of CDs from citric acid as the precursor at 180°C in air for 40 h. Underneath showing the citric acid precursor to CD–CO_2_H powder product after syringe filtration (0.45 μm) and freeze-drying.

CDs were synthesized via the thermolysis of citric acid (Scheme 1) as previously reported (see Methods for details) (Martindale et al. 2015). CD–CO_2_H was chosen as the photosensitizer for this hybrid photosystem as the carboxylic acid functionality allows for good water solubility and ease of further functionalization for future hybrid systems due to the many reactions able to readily take place with carboxylic acids (Clayden et al. 2012).

**Figure 1.**
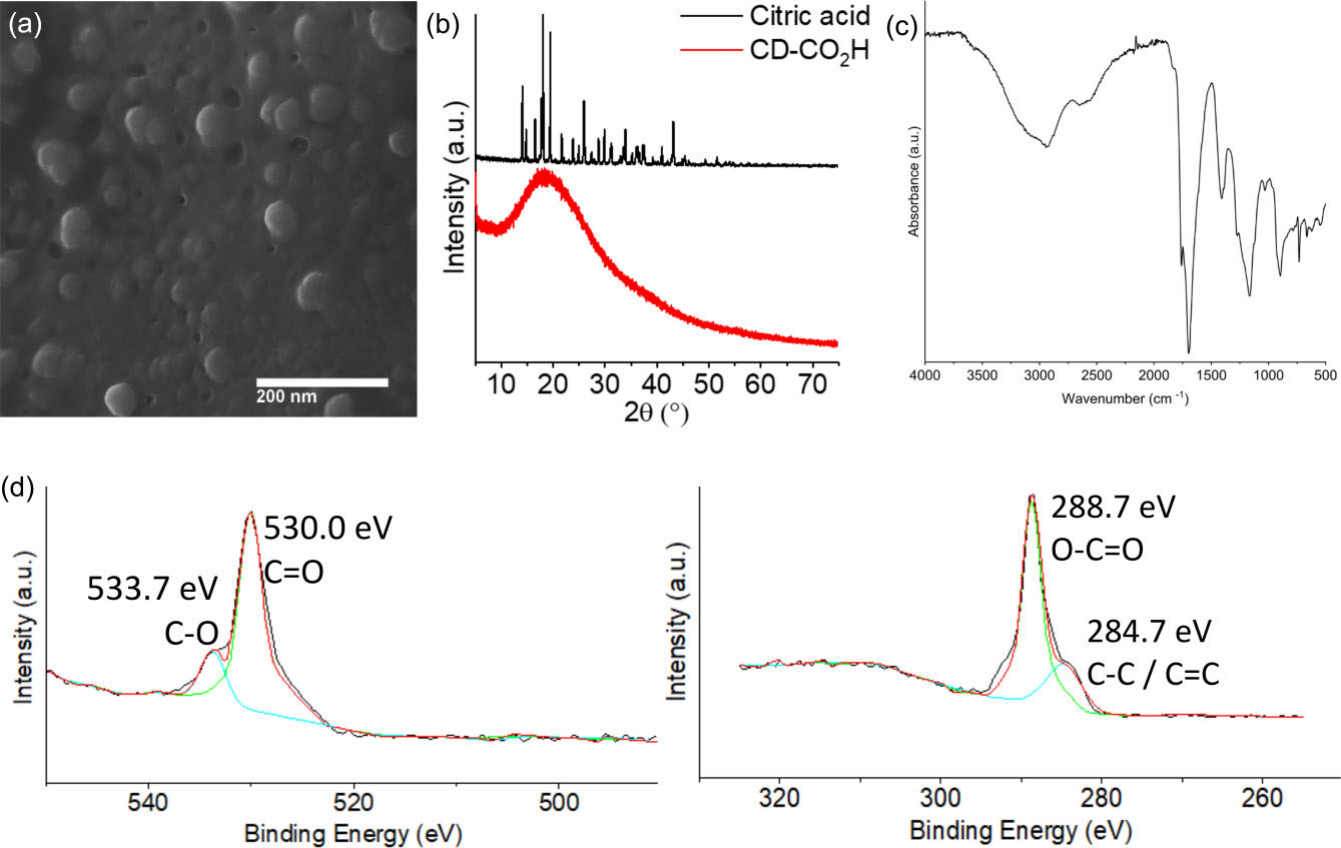
Characterization of CD–CO_2_H: (a) scanning electron microscopy (SEM) image of CD–CO_2_H; (b) X-ray diffraction pattern (XRD) of the citric acid precursor prior to heating at 180°C in air for 40 h and the XRD pattern of the resultant CD–CO_2_H after exposure to the aforementioned reaction conditions; (c) Fourier transform infrared spectroscopy (FTIR) of CD-CO_2_H powder; and (d) XPS of CD–CO_2_H showing the surface composition comprising of oxygen O1s binding energies, carbon C1s binding energies, and their corresponding bonds.

To confirm the synthesis of CD–CO_2_H, several techniques were carried out. Scanning electron microscopy (SEM) of CD–CO_2_H showed spherical particle-like structures with a moderately wide range of size (5–35 nm) (Fig. 1a). X-ray diffraction (XRD) analysis was used to compare the citric acid that was used as a precursor to the final product and a loss of crystallinity was observed (Fig. 1b). The broad peak observed at 18.5° is indicative of an amorphous substance, consistent with the nature of CDs, which are typically made of sp. (Yang et al. 2012) and sp. (Boardman et al. 1980) carbons (Tepliakov et al. 2019). Moreover, the loss of crystallinity in the substance confirms that no precursors remained in the final product.

FTIR analysis was performed to obtain information about the surface functionalities of CD–CO_2_H (Fig. 1c). The broad peak spanning between 3500 and 2150 cm^−1^ at 2937 cm^−1^ corresponds to the O–H stretching vibration. Given that CD–CO_2_H is very hygroscopic, the large span of the O–H stretching vibration was expected. Within this, there is a small peak at 2604 cm^−1^ associated with the stretching vibrations of C–C bonds and the bending vibration of C–H. The strong, sharp peak at 1760 and 1694 cm^−1^ is assigned to the C=O stretching vibrations. The peaks at 1406 and 1158 cm^−1^ are associated with C–O–H in-plane bending and C–O stretching vibrations, respectively. The stated values are well within the range of literature values (Smith 2018, Ren et al. 2019). Given that the C=O and C–O–H peaks are observed in the FT–IR spectra, full carbonization of the citric acid precursor did not take place. The carboxylic functionality that remains therefore can be exploited in the future and allow for further reactions to take place that will result in differently functionalized CDs.

XPS was used to determine the composition of the CD–CO_2_H powder and the overall XPS pattern is shown in Supplementary Fig. S1. Figure 1d revealed two peaks at 530.0 and 288.7 eV, which are attributed to O1s and C1s, respectively. The O1s spectra shown in Fig. 1d show peaks at 530.0−533.7 eV, which are attributed to C=O and C−O, respectively, owing to the carboxylic acid functionalization in the CDs. The C1s spectra (Fig. 1d) showed two peaks at 288.7 and 284.7 eV, which corresponds to O−C=O and C–C/C=C bonds found in CD–CO_2_H. UV-vis spectrum of CD–CO_2_H in aqueous solution (Supplementary Fig. S2) showed an absorption band typically assigned to π−π* and n−π* absorptions (C=C and C=O, respectively) as seen in other reported CDs (Xu et al. 2017, Jiang et al. 2020, Meierhofer et al. 2020).

## Electron transfer assay

**Figure 2.**
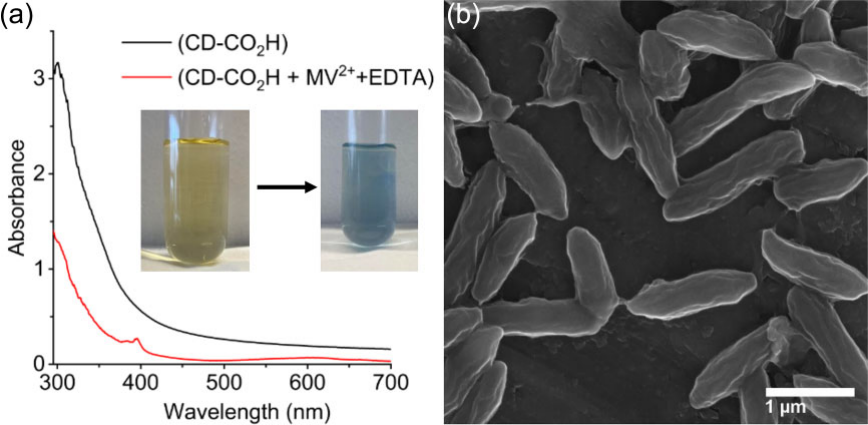
(a) UV-vis spectra of 0.5 mg/mL CD–CO_2_H with methyl viologen (1.2 mM) in EDTA (0.1 M) at pH 6 before and after exposure to solar irradiation (AM 1.5 G, I = 100 mW cm^−2^) at 25°C for 5 min minimum. Characterization of pure *G. sulfurreducens* cultures (b) SEM of *G. sulfurreducens* cells.

To investigate the electron transfer suitability of CD–CO_2_H and establish whether the synthesized CD would be a suitable light absorber, a solution containing CD–CO_2_H (1.5 mg mL^−1^), methyl viologen (MV^2+^ 1.2 mM), and EDTA (0.1 M) at pH 6 was bubbled with N_2_:CO_2_ (80%:20%) for 30 min to remove oxygen. UV-vis of CD–CO_2_H in distilled water (dH_2_O) showed an absorption peak at 298 nm (Fig. 2a) (Fang et al. 2020). Within 5 min of solar irradiation exposure, the solution turned from yellow to blue (inset Fig. 2a). This, along with the appearance of a small peak at 383 nm followed by a larger peak at 395–605 nm in the UV-vis spectra shown in Fig. 2a, is indicative of the formation of MV^+•^, the reduced species of MV^2+^. This is in agreement with previous observations with the UV-vis spectra of MV^+•^ obtained in other reported works (Watanabe and Honda 1982, Walia et al. 2019a, Ladomenou et al. 2021). Thus, CD–CO_2_H is a suitable light absorber with befitting electron transfer properties that will aid in the electron transfer process. Aside from this, the formation of the radical appears to be reversible, as removing the solution from solar irradiation resulted in the solution reverting back to its original yellow colour. The colour change continued after repeatedly exposing the solution to light and dark conditions, suggesting that CD–CO_2_H does not degrade after being exposed to solar irradiation for a long time.

## Characterization of *G. sulfurreducens*

Prior to the photoexperiment, *G. sulfurreducens* was characterized using both TEM (Supplementary Fig. S3) and SEM (Fig. 2b). Both images show the characteristic rod-shaped cell morphology (up to 2 μm in length and 0.5 μm in width). Supplementary Fig. S3 (white arrow) shows e-PNs emanating from *G. sulfurreducens* with ∼3 nm in diameter and several μm in length. *G. sulfurreducens* uses these e-PNs to transport electrons in/out of the cells. UV-vis spectrum of *G. sulfurreducens* (Supplementary Fig. S4) showed a peak at 298 nm in association with the pink hue of the microbe stemming from heme (Fe) containing c-type cytochromes (Fang et al. 2020).

## Photoexperiment

**Figure 3.**
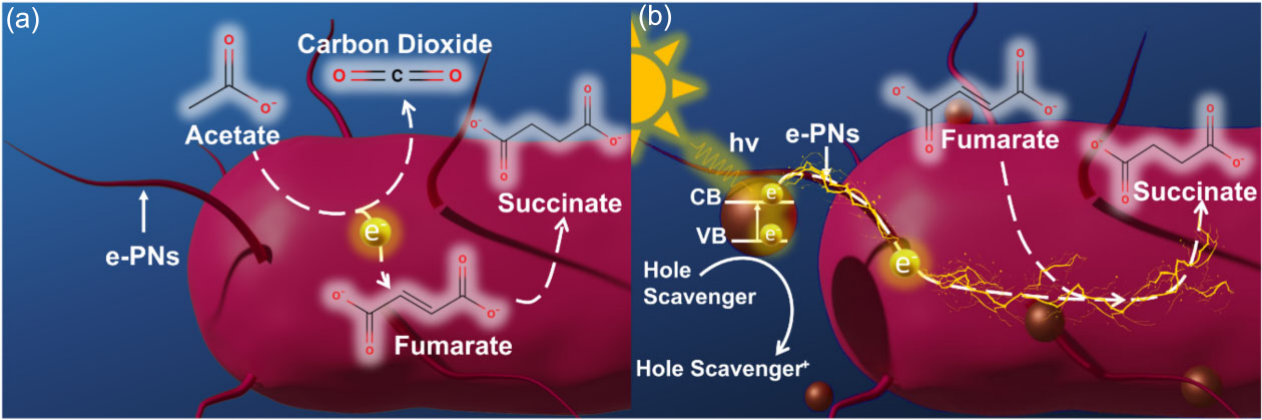
Mechanistic representation of the traditional fumarate reduction with only *G. sulfurreducens* with energy-rich acetate as the electron donor and the semi-biological photosystem developed in this work. (a) Traditionally, *G. sulfurreducens* uses acetate as the electron donor and will absorb it indiscriminately throughout the cell, resulting in the reduction of fumarate to succinate. (b) In this work, *G. sulfurreducens* was introduced to a solution containing CD–CO_2_H, fumarate, and triethanolamine (TEOA) as the hole scavenger for the two electron reduction (hydrogenation) of fumarate to succinate. Here CDs act as the electron donor for *G. sulfurreducens* instead of acetate. The c-type cytochromes-based e-PNs act as a conduit for the electrons to be transported from the CD to the relevant metabolic cascades within *G. sulfurreducens*.

Traditionally, to culture *G. sulfurreducens*, energy-rich acetate is used as the electron donor, and fumarate accepts the metabolically generated electrons and undergoes reduction to succinate (Fig. 3a). However, in our semi-biological system, acetate is replaced by CD–CO_2_H as the sustainable electron donor (Fig. 3b). The photocatalytic performance of the CD−*G. sulfurreducens* biohybrid was studied under solar light irradiation in the presence of fumarate as the electron acceptor, TEOA as the sacrificial hole scavenger, and CDs as the potential electron donors instead of energy-rich acetate. CD–CO_2_H (1.5 mg/mL) was dissolved in a modified DSMZ 826 *G. sulfurreducens* growth media (15 mL, pH = 7; see Supplementary Table S3 for more details) along with sodium fumarate (50 mM) and TEOA (20 mM). After purging the vial with N_2_:CO_2_ for 45 min, *G. sulfurreducens* was added to the photoreactor vial (optical density OD_600_ = 0.70). The photo-vial was then irradiated with solar light (AM 1.5 G, 1 sun, 100 mW cm^−2^) for the duration of the experiment. The experiments were performed at room temperature and neutral pH under gentle stirring.

**Figure 4.**
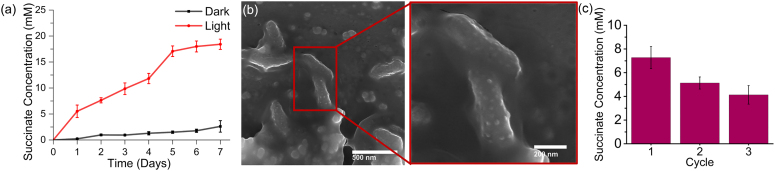
CD–CO_2_H integrated with *G. sulfurreducens* in the presence of TEOA as the hole scavenger for the photocatalytic reduction of fumarate to succinate. (a) Production of succinate over 7 days under light and dark conditions. The conditions are as follows: 25°C, 150 revolutions per min, 50 mM fumarate, 20 mM TEOA, 1.5 mg/mL CD–CO_2_H, OD_600_ = 0.70, AM 1.5 G, and 100 mW cm^−2^. (b) SEM of pure *G. sulfurreducens* culture after the photoexperiment alongside a magnified image of the highlighted section showing CDs on the surface of *G. sulfurreducens*. (c) Stability test for the production of succinate by the CD–*G. sulfurreducens* hybrid photosystem over three cycles (each cycle lasting 3 days each). CDs and *G. sulfurreducens* in cycles two and three were reused from their previous cycles and the experiments were performed in triplicate. The conditions were as follows: 25°C, 150 revolutions per min, 50 mM fumarate, 20 mM TEOA, 1.5 mg/mL CD–CO_2_H, OD_600_ = 0.70, AM 1.5 G, and 100 mW cm^−2^.

We monitored succinate production over time using ^1^H NMR. Figure 4a shows that under light conditions, the CD−*G. sulfurreducens* hybrid produces greater quantities of succinate (7-fold) compared to that under dark conditions. After 5 days, succinate production appeared to have decreased, most likely stemming from the diminishing quantity of fumarate (the electron acceptor) in the photovial. The inactivity of the CDs in the dark reaction along with the low concentration of succinate produced demonstrates the vital role of CDs in contributing to the enhanced production of succinate. This is also observed in the control experiment where no CDs were added to the system, and as a consequence, much lower quantities of succinate were yielded (Supplementary Fig. S5). Also, without the addition of *G. sulfurreducens* to the hybrid system, there was no biological catalyst to reduce fumarate to succinate, and thus, no succinate was detected (Supplementary Fig. S5). SEM confirmed the integration of CDs onto the surface of *G. sulfurreducens* (Fig. 4b). The proposed mechanism between the interface of CD–CO_2_H and *G. sulfurreducens* is depicted in Fig. 3b. Upon light absorption, the electron is photoexcited from the valence band (VB) to the conduction band (CB) of the CD–CO_2_H. The e-PNs (previously identified in Supplementary Fig. S3) then transported the electrons to within *G. sulfurreducens*, where the two-electron reduction of fumarate took place. The hole scavenger, TEOA, filled the hole left behind by the photoexcited electron by donating its electrons, thus preventing recombination from occurring. A previous study of CD–CO_2_H noted that the conduction band edge is approximately −1.98 V vs. SHE (Ladomenou et al. 2021), whereas the e-PNs within *G. sulfurreducens* were discovered to have a charge of −0.19 V vs. SHE (Magnuson et al. 2001, Bond and Lovley 2003). As the e^_^PNs are more positive, the electrons are likely to be shuttled from the CD–CO_2_H light absorber to the *G. sulfurreducens*.

There was a small amount of succinate production under dark conditions (Fig. 4a), as *G. sulfurreducens* can use stored carbon during its growth with acetate as a mechanism to cope with the starving conditions (Mason-Jones et al. 2023) it was subjected to as part of the preparation for the photoexperiment. Microbes often prioritize survival over growth and maintenance, especially under nutrient-limiting conditions (Merchant and Helmann 2012). As such, *G. sulfurreducens* stored an amount of acetate to act as an emergency reserve so even in the dark experiment without acetate, *G. sulfurreducens* was able to use the stored acetate it accumulated prior to produce small amounts of succinate.

Earlier work on *G. sulfurreducens* disclosed that the catalytic performance of said microbe improved under visible light and that the light-induced bacterial electron transfer correlated well with rates of substrate consumption and microbial respiration (Li et al. 2014). Postulating that the cytochrome nanowires may be photoactive, Neu et al. (2022) quantified the photoresponse of individual e-PNs using a mutated *G. sulfurreducens* strain, *G. sulfurreducens* CL-1, which overexpressed OmcS nanowires and revealed that under photoexcitation, e-PNs showed up to 100-fold conductance. Thus, in our CD−*G. sulfurreducens* photosystem, solar irradiation perhaps not only photoexcited the electrons in CDs but also further increased the conductance of e-PNs for extracellular electron transport from CDs to *G. sulfurreducens*, greatly contributing to the overall efficiency of the fumarate reduction compared to dark conditions. This would need to be confirmed via conductance studies under solar irradiation and compared to dark conditions.

The artificial photocatalytic reduction of fumarate to succinate on the surface of sphalerite colloids had been carried out in a previous study (Zhou and Guzman 2016). However, further studies into this system revealed that fumarate adsorption was found to be highly pH-dependent (Mangiante et al. 2019). A prior study used the enzymes present in *Shewanella oneidensis* MR-1 as the biological catalyst and eosin Y as the light absorber for fumarate reduction (Rowe et al. 2017). Only trace amounts of succinate could be detected (<0.01 mmol), whereas in our system, by using qNMR, we calculated to have obtained ∼0.2 mmol in the same amount of time. However, a direct comparison of the hybrid systems is not possible, as it must be noted that Rowe’s study used a greater amount of TEOA, and in our study, a greater quantity of fumarate was used. Moreover, methyl viologen was used as a redox mediator in their system. Methyl viologen is known to be able to permeate the cell membranes and shuttle electrons to the relevant enzymes. However, along with other artificial electron shuttles, it is considered toxic to microbes even at low concentrations (Yonei et al. 1986, Castro et al. 2008) and can hinder the microbe’s ability to conserve energy for growth and cell maintenance (Lovley 2006). This will negatively affect the longevity of the system in the long term and cause unnecessary side reactions, which are especially undesirable if implemented in industry. The c-type cytochromes and e-PNs from *G. sulfurreducens* in our semi-biological hybrid system compensate for this, eliminating the need for such toxic redox mediators. In using a microbe instead of an enzyme like in the above study, our semi-biological photosystem is also more forgiving in the range of pH that it can operate. Unlike the pH-dependent system involving sphalerite colloids where 50% of fumarate was absorbed at pH 5.4 (Mangiante et al. 2019), fumarate is a natural electron acceptor for *G. sulfurreducens* and thus readily takes it in as part of its metabolic cycle.

One of the advantages of using CDs instead of traditional semiconductors, such as cadmium sulphide (CdS) and platinum quantum dots, is that they do not use any precious metals and are low in toxicity. A solar-driven semi-biological photosystem involving the use of *Moorella thermoacetica* and CdS for CO_2_ conversion resulted in Cd^2+^ precipitation upon solar irradiation (Göbbels et al. 2021), whereby it risks interference with the biocatalytic processes of the microbial cells and leads to toxic effects on the cells, thus affecting the sustainability of the CO_2_ conversion. Cadmium selenide (CdSe) quantum dots, another nanomaterial containing toxic elements, were coupled with platinum and poly (3,4-ethylenedioxythiophene−poly(styrenesulfonate) (PEDOT:PSS) for solar-driven hydrogen production (Li et al. 2018). Unlike our photosystem, where a neutral pH was used, an acidic electrolyte (pH = 1) was required for hydrogen evolution to be favoured. CdSe itself was shown to be susceptible to photocorrosion and the addition of Pt and PEDOT:PSS alleviated it. The photo-instability of CdS is also demonstrated in the work by El-Maghrabi et al. (2018), where TiO_2_ has to be used as a coating to improve photocorrosion. However, it was also suggested that sulphides or sulphur trioxide be added as an SED to the system, which would only increase the amount of toxic components within the system and make disposal of the waste even more complicated. On the other hand, in our semi-biological photosystem, our CDs are less susceptible to photocorrosion and can be reused. As depicted in Fig. 4c, three cycles of solar irradiation were subjected to the same semi-biological photoreactor. A minimum of 70% succinate was recovered from each cycle, indicating that both the CDs and *G. sulfurreducens* were still viable and could be reused. This demonstrates the durability, stability, and reusability of the photosystem where, potentially, for long-term use, only the hole scavenger and electron acceptor will need to be replenished.

Toxicity aside, other reported photosystems focused on the reduction of CO_2_. Limited by the wide bandgap of TiO_2_, the solar-to-methane conversion efficiency of the semi-biological photoelectrochemical system developed by Fu et al. (2018) was reported to be only 0.1%. In comparison, Xiao et al. (2020) achieved 1.28% solar conversion efficiency. A major downside to these systems is that the product obtained is methane, a greenhouse gas. Although it can be valuable in industry, it is still considered a greenhouse gas that contributes to global warming, a phenomenon that several global initiatives have been formed to combat (Rose et al. 2017, Wang et al. 2022). Our work reports the hydrogenation of fumarate to succinate, which is more sustainable as it is not considered a greenhouse gas, it can be performed under atmospheric temperature and pressure, and it can be used to synthesize a variety of products through reactions requiring milder conditions. For example, producing γ-butryolactone through hydrogenation only requires up to 175°C (Werpy et al. 2005), and esterification over ion-exchange resins has been reported to have proceeded at temperatures ranging from 80 to 120°C (Kolah et al. 2008). Thus, our semi-biological system is a lot less burdensome on the earth’s resources and is more sustainable.

### Future perspective: economic considerations for future scalability

Scaling our semi-biological hybrid photosystem from a laboratory setting to an industry scale is very challenging. Traditionally, the synthesis of succinate involved the use of petrochemicals, but due to the high oil prices back in 2007 (Pinazo et al. 2015), many ventures such as BioAmber and Reverdia built plants for the commercial production of succinic acid (Ebert 2007). Narisetty et al. (2022) found the production costs of petroleum and bio-based succinic acid to be €2554/megatonne and €1045/Mt, respectively, showcasing the competitiveness of bio-based succinic acid. Despite unreleased patents and information not being publicly made available, there are some aspects we are able to consider. For example, looking at the fermentation process to make succinic acid, the analysis undertaken by Morales et al. (2016) 10–15% of the total production costs go to feedstock purchases, 20–25% to fermentation processes, and 60–80% to purification processes. Although our hybrid system eliminates the need for fermentation, the financial cost of purification is still an issue. Compared to industries such as BioAmber and Reverdia, where high temperatures are involved in the fermentation process (Shekiro et al. 2014, Salvachúa et al. 2016, Mancini et al. 2022), our hybrid photosystem requires only an ambient temperature and thus is much less energy, and therefore cost-intensive. Equipment costs are also significant; Reverdia, BioAmber, and Yedur et al. all required fermenters, multiple evaporation and filtration units, crystallizers, and acid-resistant equipment (Mancini et al. 2022), all of which drive up the cost of production as well as maintenance. Likewise, scaling our system up will also require similar sophisticated equipment, raising costs.

Overall, it is important to note that compared to an industrial process, our hybrid system developed on a bench is much less refined, and, taking equipment, reagents, energy costs, etc. into account, scaling up will undoubtedly increase the expense. Nevertheless, understanding the factors influencing the economic landscape, including the commercialization prospects, cost drivers, and the demand for succinic acid, is vital for the commercialization of not just bio-synthesized succinic acid but also other biofuels.

## Conclusion

In summary, this study provides proof-of-concept of CDs being able to successfully interface with *G. sulfurreducens*. Enhanced succinate production was observed under solar irradiation, indicating that the photo-excited electrons generated under light conditions were able to be taken by *G. sulfurreducens* to reduce fumarate to succinate. Compared to the aforementioned hybrid system involving *S. oneidensis* MR-1 and eosin Y (Rowe et al. 2017), our system produced greater amounts of succinate in the same amount of time without the need for a redox mediator. This demonstrates not only good compatibility between *G. sulfurreducens* and CDs, but also that the e-PNs emanating from *G. sulfurreducens* cells can act as a wire to facilitate the electron transfer between the two components, enabling us to circumvent the use of redox mediators (which are often toxic and expensive) and help overcome the sluggish electron transfer kinetics. Although it has been postulated that the e-PNs facilitate this electron transfer, additional work will need to be carried out to confirm this, in particular using the adapted strain *G. sulfurreducens* KN400, which is said to be even more effective in extracellular electron transfer than *G. sulfurreducens* due to the greater number of expressed e-PNs (Yi et al. 2009). Nevertheless, the semi-biological hybrid photosystem developed in this work stands as a proof of concept that interfacing CDs with *G. sulfurreducens* will exhibit enhanced succinate production under light conditions without the need for a redox mediator. Moreover, the ambient conditions needed, the non-toxic, reusable, and cheap CDs, and the ability of succinate to be processed further to higher-value products under mild conditions (compared to those needed for processing methane obtained from CO_2_ reduction), attest to the sustainability of our system.

## Supplementary Material

qvae020_Supplemental_File

## Data Availability

The data in this article is available in this article and in its online supplementary material.
