# Supplementary material for: Semi-biological photosystem: harnessing carbon dots and Geobacter sulfurreducens for solar-driven hydrogenation
Source: Sustain Microbiol. 2024 Jul 22;1(1):qvae020. doi: 10.1093/sumbio/qvae020 (PMC13375293; doi:10.1093/sumbio/qvae020)
Supplement: Supplementary file 1 [file qvae020_Supplemental_File.pdf]

Supporting Information

Semi−biological photosystem: Harnessing carbon dots and *Geobacter sulfurreducens* for solar−driven hydrogenation

Mandy Ching Man Yau[1] and Shafeer Kalathil[1*]

[1]Hub for Biotechnology in the Built Environment, Department of Applied Sciences, Faculty of Health and Life Sciences, Northumbria University, Newcastle, NE1 8ST, United Kingdom

*Corresponding author: shafeer.kalathil@northumbria.ac.uk

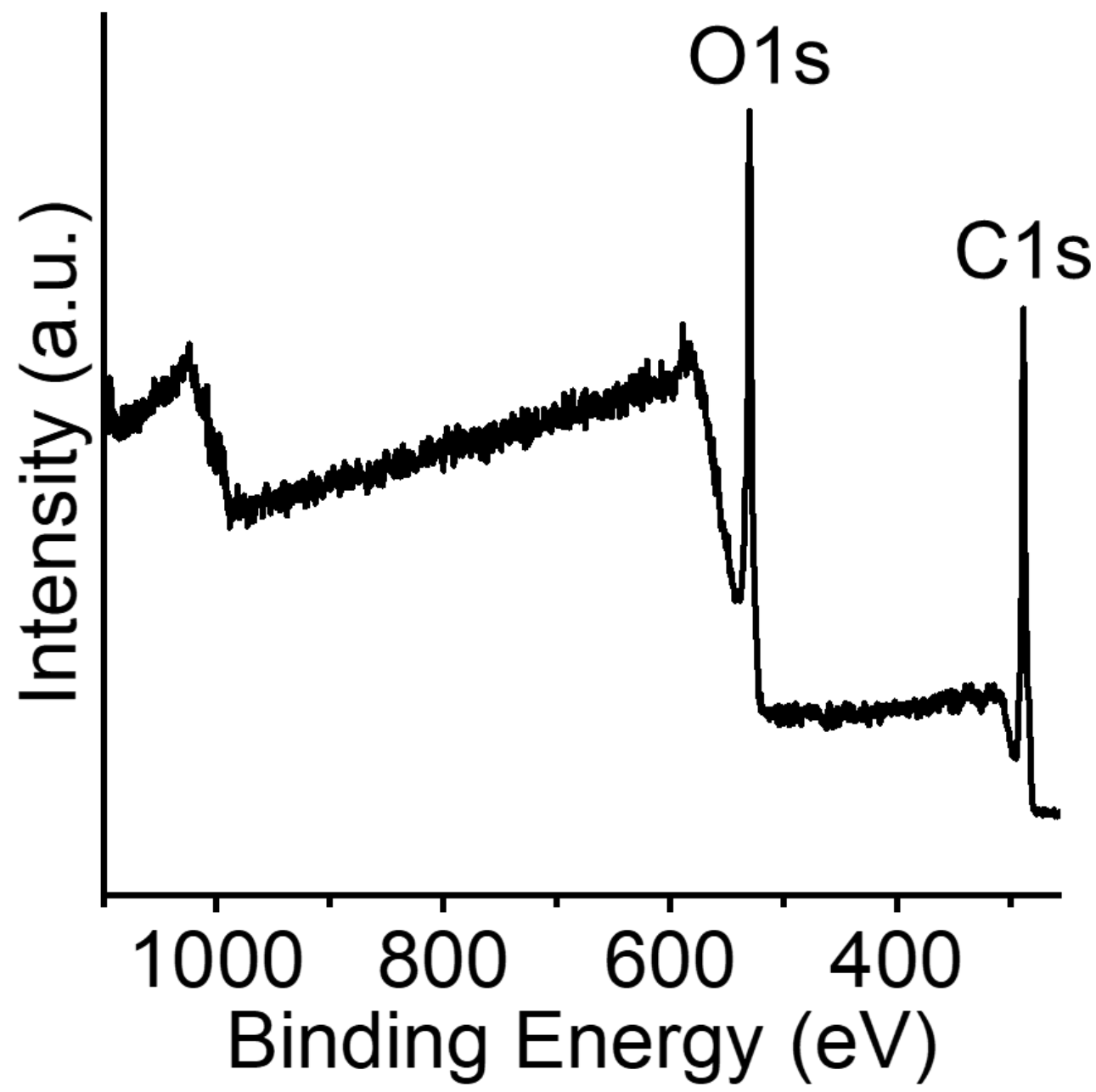

Figure S1: XPS survey spectra for CD-CO_2H

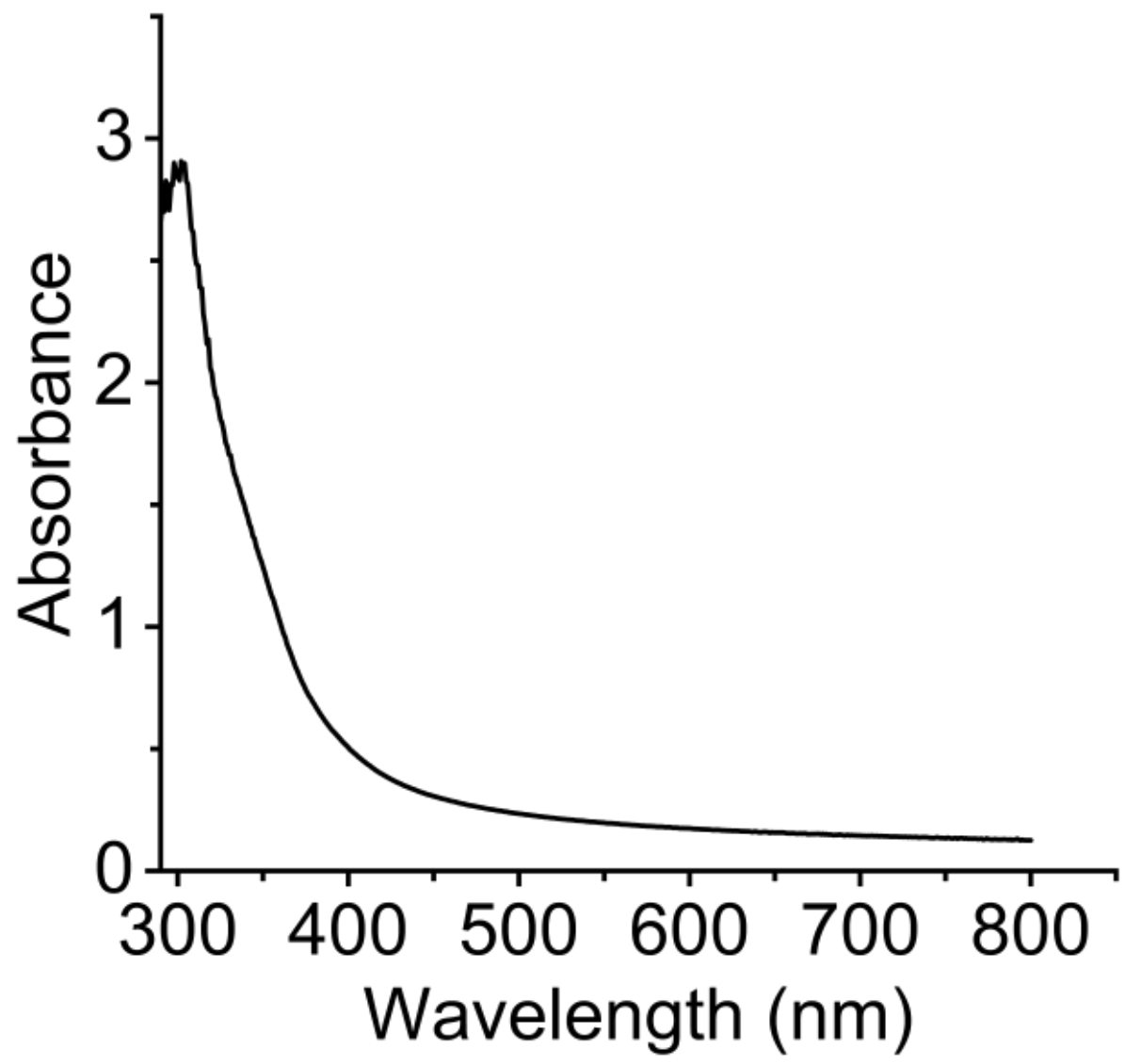

Figure S2: UV-vis absorption spectra of 0.5 mg/mL CD-CO_2H in dH_2O at 25 °C. The path length of the cuvette was 1 cm.

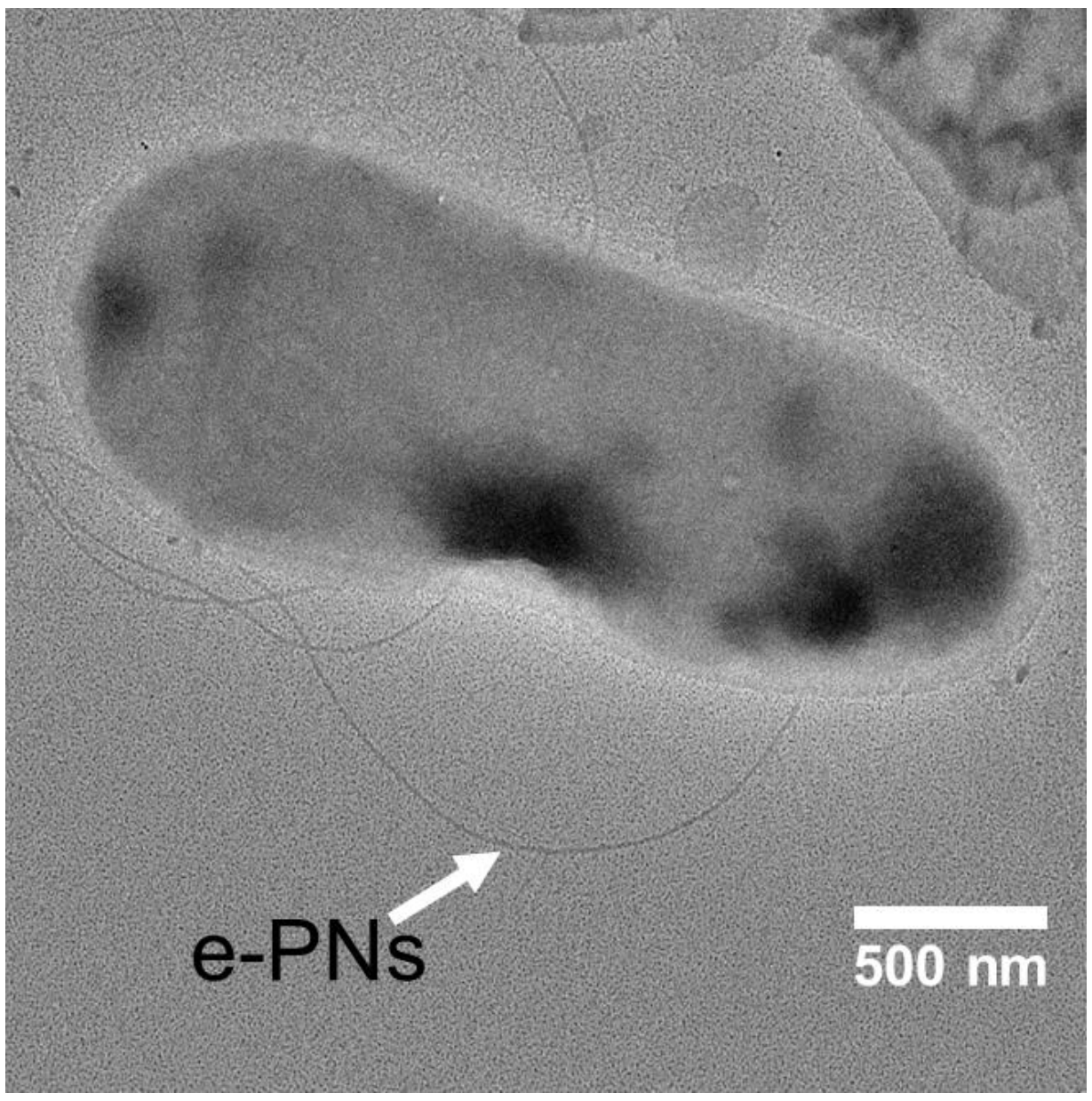

Figure S3: Transmission electron microscopic (TEM) image of a single G. sulfurreducens cell with the electrically conductive protein nanowire (e−PNs) emanating from the cell (indicated by the white arrow).

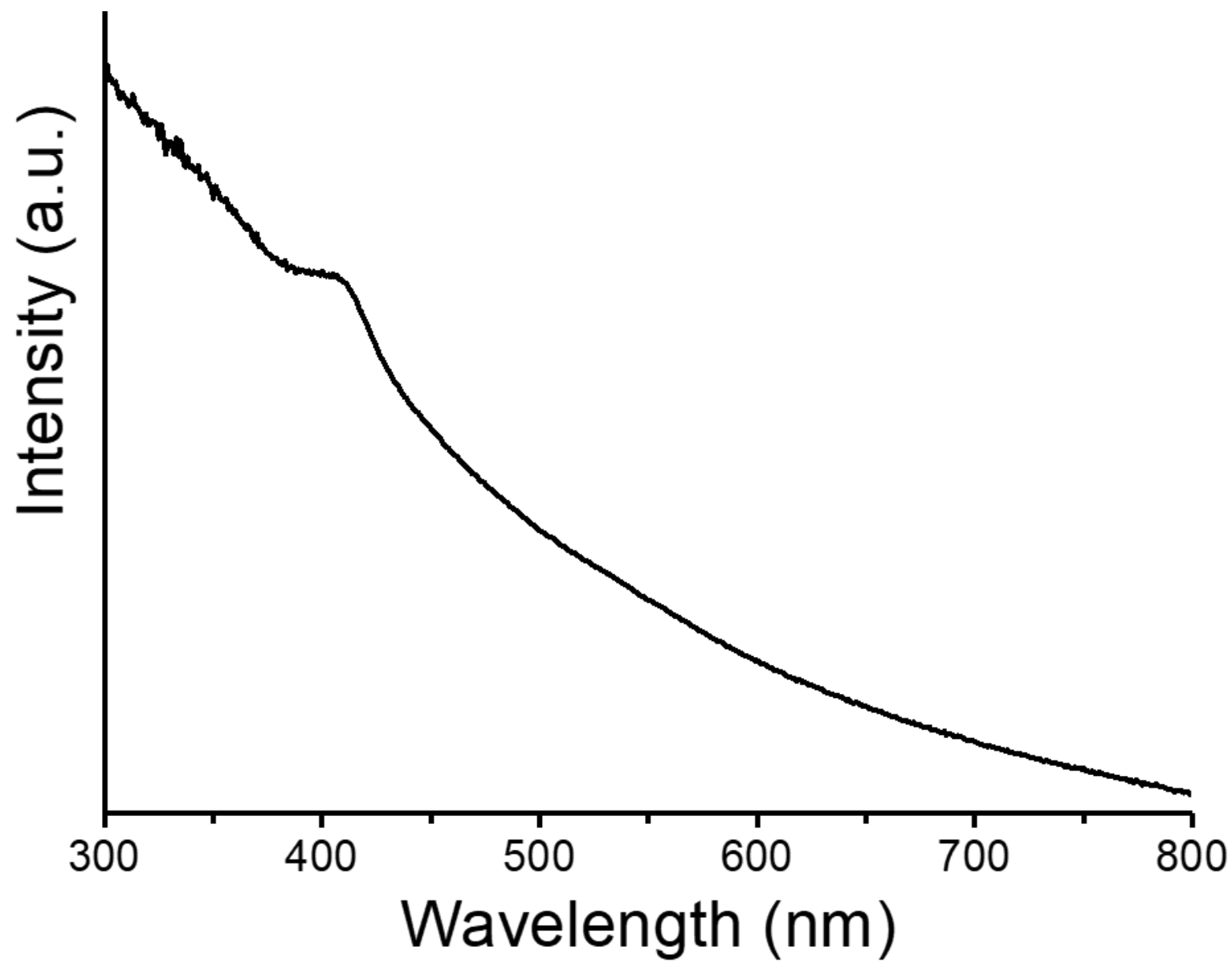

Figure S4: UV-vis of *G. sulfurreducens* (OD_{600} = 0.70)

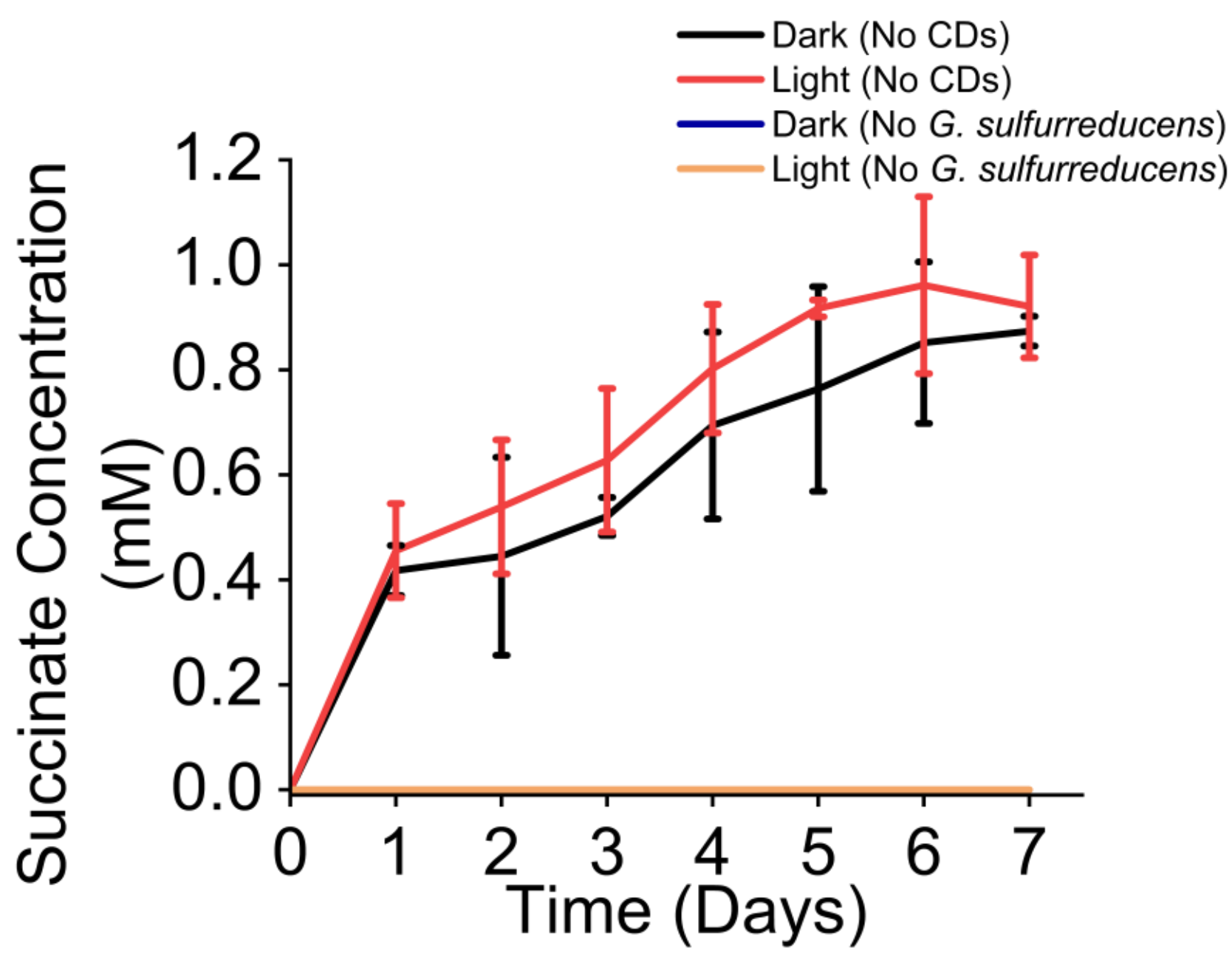

Figure S5: Control experiments for *G. sulfurreducens* production of succinate under light and dark conditions and cell-free (i.e. *G. sulfurreducens* free) experiments under light and dark conditions, both conducted over a 7-day period. The conditions are as follows: 25 °C, 150 revolutions per minute, 50 mM fumarate, 20 mM TEOA, AM 1.5G, 100 mW cm^{-2}.For the experiment with no CDs, *G. sulfurreducens* was added until OD_{600} = 0.70. For the cell-free experiment, 1.5 mg/mL CDs was added.

Table S1: Trace element solution (from DSMZ medium 141)

Wolin's mineral solution (one litre) was made according to the DSMZ from medium 141.

Chemical	Amount
Nitrilotriacetic acid	1.50 g
$MgSO_4 \times 7H_2O$	3.00 g
$MnSO_4 \times H_2O$	0.50 g
NaCl	1.00 g
$FeSO_4 \times 7H_2O$	0.10 g
$CoSO_4 \times 7H_2O$	0.18 g
$CaCl_2 \times 2H_2O$	0.10 g
$ZnSO_4 \times 7H_2O$	0.18 g
$CuSO_4 \times 5H_2O$	0.01 g
$AlK(SO_4)_2 \times 12H_2O$	0.02 g
H_3BO_3	0.01 g
$Na_2MoO_4 \times 2H_2O$	0.01 g
$NiCl_2 \times 6H_2O$	0.03 g
$Na_2SeO_3 \times 5H_2O$	0.30 mg
$Na_2WO_4 \times 2H_2O$	0.40 mg
dH_2O (autoclaved)	1000 mL

Table S2: Vitamin solution (from DSMZ medium 120)

Wolin's vitamin solution (one litre) was made according to the DSMZ from medium 120).

Chemical	Amount
Biotin	20.00 mg
Folic acid	20.00 mg
Pyridoxine hydrochloride	100.00 mg
Thiamine×HCl	50.00 mg
Riboflavin	50.00 mg
Nicotinic acid	50.00 mg
Calcium D-(+)-pantothenate	50.00 mg
Vitamin B12	1.00 mg

p-Aminobenzoic acid	50.00 mg
(DL)-a-Lipoic acid	50.00 mg
dH2O (autoclaved)	1000 mL

Table S3: Microbial medium 826 (fumarate and acetate omitted)

The culture media (one litre) for *G*. *sulfurreducens* was made according to DSMZ, albeit with sodium fumarate and sodium acetate omitted.

**Chemical**	**Amount**
NH_4Cl	1.50 g
Na_2HPO_4	0.60 g
KCl	0.10 g
dH_2O (autoclaved)	1000 mL

The solution was placed autoclaved before Wolin's mineral solution (10 mL), $NaHCO_3$ (2.50 g), and Wolin's vitamin solution (1.00 mL) was added.
